# Activated Serum Increases In Vitro Cellular Proliferation and Growth Factor Expression of Musculoskeletal Cells

**DOI:** 10.3390/jcm11123442

**Published:** 2022-06-15

**Authors:** Owen P. Karsmarski, Benjamin C. Hawthorne, Antonio Cusano, Matthew R. LeVasseur, Ian J. Wellington, Mary Beth McCarthy, Mark P. Cote, Augustus D. Mazzocca

**Affiliations:** Department of Orthopaedic Surgery, University of Connecticut, Farmington, CT 06032, USA; bhawthorne@uchc.edu (B.C.H.); acusano@uchc.edu (A.C.); mlevasseur@uchc.edu (M.R.L.); iwellington@uchc.edu (I.J.W.); mccarthy@uchc.edu (M.B.M.); mcote@uchc.edu (M.P.C.); mazzocca@uchc.edu (A.D.M.)

**Keywords:** thrombin, autologous activated serum, activated serum, osteoblasts, tenocytes, bursa, chondrocytes, orthobiologics

## Abstract

The purpose of this study was to investigate proteomic alteration that occurs to whole blood when converted to activated serum (AS) using an autologous thrombin system. This study further sought to evaluate the functional in vitro effect of AS on tenocytes, chondrocytes, subacromial bursal cells, and osteoblasts. The peptide/protein composition of AS was analyzed by liquid chromatography–mass spectrophotometry (LC-MS). The cell lines were treated with AS, and cellular proliferation was quantified 48 h after treatment. Platelet-derived growth factor (PDGF), insulin-like growth factor 1 (IGF-1), vascular endothelial growth factor (VEGF), interleukin-1 beta (IL-1β), and interleukin-1 receptor antagonist (IL-1Ra) were quantified utilizing enzyme-linked immunosorbent assays (ELISAs). LC-MS identified 357 proteins across the AS and whole blood. Fifty-four of the proteins identified had significant differences between the relative protein abundance of the AS samples compared to whole blood. Treatment with AS in all cell lines significantly increased proliferation compared to control cells at 48 h. Increased PDGF, VEGF, and IGF-1 in all cell lines exposed to AS compared to the control (*p* < 0.05) were observed. These findings suggest that treatment with AS increases in vitro cellular proliferation and the release of growth factors that may play a role in tissue repair.

## 1. Introduction

Orthobiologics is an emerging field exploring augments to standards of care and new treatment modalities for orthopedic surgeries [1,2,3,4,5,6]. A new biologic modality uses point-of-care devices to convert a small quantity of whole blood into autologous activated serum (AAS) [7]. AAS has a higher concentration of thrombin than whole blood, allowing for its use in creating autologous clots. The use of autologous thrombin has taken the place of bovine and other non-autologous thrombin, making it safer for use in humans [8,9]. However, AAS is not isolated thrombin and potentially contains other beneficial biologic growth factors and cytokines [7]. Autologous blood products, such as platelet-rich plasma (PRP) and autologous-conditioned serum, have previously demonstrated promise for biologic augmentation [10,11,12]. PRP is a serum that is derived from autologous plasma that requires centrifugation and contains growth factors implicated in healing processes throughout the body [13,14]. Autologous-conditioned serum is created by incubating whole blood at 37 °C in the presence of glass beads to increase anti-inflammatory cytokines [15,16]. Autologous thrombin systems were developed to rapidly create thrombin during surgical operations by activating the coagulation cascade through calcium chloride and negatively charged glass beads [17]. Compared to PRP and autologous-conditioned serum, these devices provide the advantage of not requiring centrifugation and long incubation periods, thus providing an added benefit in point-of-care application [17].

Through the activation of the coagulation cascade, the autologous thrombin systems potentially trigger the release of hundreds of proteins that modulate cellular proliferation and wound healing. Some of these endogenous factors include platelet-derived growth factor (PDGF), vascular endothelial growth factor (VEGF), and connective tissue growth factor (CTGF), as well as inflammatory modulators such as IL-β, among others, stored and produced by the activated human platelet [7,18,19]. The purpose of this study was to investigate the proteomic alteration that occurs when whole blood is activated using an autologous thrombin system. This study further evaluated the functional in vitro effect of activated serum (AS) on human musculoskeletal cells. The authors hypothesized that AS retrieved from the autologous thrombin system would alter the relative protein abundance compared to whole blood, and that treating cell lines would increase cellular proliferation and protein expression.

## 2. Materials and Methods

### 2.1. Preparation of Activated Serum

Three different 100 mL samples of human whole blood (*n* = 3) were obtained (LAMPIRE Biological Laboratories Inc, Pipersville, PA, USA). According to the manufacturer’s protocol, AS was isolated from whole blood using the Thrombinator^®^ (Arthrex Inc., Naples, FL, USA). Amounts of 0.1 mL of calcium chloride and 4 mL of blood fraction were combined and mixed within the provided device container for five seconds. This solution was placed flat for ten minutes to allow the clot to form. The device was shaken to break the clot, and 0.2 mL of calcium chloride and 8 mL of each corresponding whole blood sample were added. The device was again mixed for five seconds and placed flat for one minute. The device was shaken a second time to break the clot. The provided filter was placed on the withdraw port, and the AS was withdrawn through the filter using a 10 mL syringe. To determine the peptide/protein composition of the three AS samples, they were prepared for liquid chromatography–mass spectrophotometry (LC-MS) and were compared to three whole blood samples as a control.

### 2.2. Liquid Chromatography-Mass Spectrophotometry Sample Preparation

The tandem use of liquid chromatography and mass spectrophotometry (LC-MS) has been used to identify the peptide and protein make-up of complex biologic fluids in organisms [20]. The LC-MS applications to blood products can be used to identify peptides and proteins through a physical separation and identifying their ratio of mass to charge via ionization of the proteins. This method allows for identifying and isolating many components within a greater, complex mixture.

After the AS was withdrawn through the device filter, the whole blood and AS samples were decellularized via centrifugation for at least 15 min at 2200–2500 rpm. The soluble decellularized component on top was removed and placed into new tubes. Protein concentrations for each sample were determined using the Pierce^TM^ Protein bicinchoninic acid (BCA) assays (Thermo Fischer Scientific, Waltham, MA, USA) to determine the protein concentrations. BCA assays were run following the protocol supplied by Thermo Fischer Scientific. Absorbance values were obtained using the BioTek^®^ 96-well microplate reader (BioTek, Winooski, VT, USA) measuring absorbance values of 562 nm. Five hundred micrograms of protein from each sample was incubated while mixing with High-Select^TM^ Top 14 Abundant Protein Depletion Resin (Thermo Fischer Scientific, Waltham, MA, USA) within mini-spin columns for 10 min at room temperature. This depletion kit eliminates the most abundant blood components that would otherwise crowd out the signal of the less abundant proteins of interest. The proteins that were depleted from the samples included albumin, immunoglobulins (A, D, E, G, G-light chain, and M), alpha-1-acid glycoprotein, alpha-1-antitrypsin, alpha-2-macroglobulin, apolipoprotein A1, fibrinogen, haptoglobin, and transferrin. The protein depletion was necessary to increase the likelihood of identifying less abundant proteins. The mini-spin columns were then placed in 2-milliliter collection tubes, and samples were removed from the resin via centrifugation for 2 min at 1000 times gravity. A post-protein depletion BCA assay was run to determine protein concentrations after protein depletion. Prior to loading the samples into the liquid chromatography column, the samples were diluted; cysteine residues were subject to reduction and subsequently alkylated. The remaining proteins in solution were trypsinized at a ratio of 1 part trypsin to 20 parts protein while shaking for 16 h at 37 °C. The digestion was quenched after 16 h, and salt was removed from the digested sample using the Pierce C18 Peptide Desalting Spin Columns (Thermo Fischer Scientific) and the manufacturer’s instructions. See Supplemental Methods liquid chromatography-mass spectrophotometry (LC-MS) (Supplementary Materials) for a more detailed description of sample preparation prior to LC-MS [21]. A Thermo Scientific Ultimate 3000 RSLCnano UPLC system coupled directly to a Thermo Scientific Q Exactive HF mass spectrometer was used to analyze the peptide components within each sample. Peptides were ionized and then mass-separated to determine the specific peptide composition. Peptide and protein identification and quantification were performed using the MaxQuant software suite (v1.6.10.43), utilizing a Uniprot Homo sapien reference database. The search results were filtered to a one-percent false identification rate, and the protein and peptide–spectrum matches were uploaded into Scaffold Q + S (v5, Proteome Software, Inc., Portland, OR, USA) for data analysis. See Supplemental Methods liquid chromatography-mass spectrophotometry (LC-MS) (Supplementary Materials) for more details [21].

### 2.3. Liquid Chromatography-Mass Spectrophotometry Analysis

After the data were uploaded into the Scaffold Viewer (v5, Proteome Software, Inc.), the values were normalized based on their average precursor intensities, allowing them to be compared across samples. The three AS samples were compared with the three control whole blood samples in order to assess fold changes between their relative protein abundance. *p*-values were obtained given a two-tailed *t*-test that was run within Scaffold Viewer comparing their respective normalized average precursor intensities. Additionally, fold changes were expressed as numerical values unless samples in AS were below the limit of detection of the LC-MS, in which case they were expressed as a value of 0. If the whole blood samples were undetected or below the limit of detection of the LC-MS, then the fold changes for AS were expressed as infinite fold change.

### 2.4. Tissue Collection and Cell Isolation

Following peptide/protein identification with LC-MS, musculoskeletal cell lines of interest (primary tenocytes, chondrocytes, primary subacromial bursal cells, and primary osteoblasts) were cultured in order to analyze the functional phenotypic outcomes of AS exposure. For each cell line, an *n* = 6 was used and run in duplicates. Primary human cells were cultured from discarded tissue obtained during orthopedic primary rotator cuff repairs or reverse shoulder arthroplasty (IRB# IE-07-224-2). Primary human osteoblasts were prepared from bone fragments obtained from shoulder arthroplasty using a rongeur. Fresh human supraspinatus tendon and subacromial bursal tissue were obtained from primary rotator cuff repairs during arthroscopic debridement using Blakesley forceps. The tendon and bursa were cut into small fragments such that they could be digested in 2 mg/mL collagenase P (Sigma-Aldrich, St Louis, MO, USA) for 2.5 h at 37 °C, pelleted using a centrifuge, resuspended, and plated in Primaria culture dishes (Fisher Scientific, Agawam, MA, USA), as previously described [22,23]. The digested tissue was grown in complete culture medium containing DMEM (Gibco/Invitrogen), 10% FBS (Thermo Fischer Scientific, Waltham, MA, USA) and 0.1% penicillin/streptomycin (Thermo Fischer Scientific, Waltham, MA, USA). The human chondrocytes were purchased from Lonza Walkersville Inc. (Lonza Walkersville Inc., Walkersville, MD, USA). The cells were cultured in 3D-alginate beads according to the manufacturer recommendations and grown using Chondrocyte Growth Medium BulletKit^TM^ (Lonza Walkersville Inc.). All of the samples were grown in Primaria 100 mm dishes (Thermo Fischer Scientific) for three weeks or until the cells became confluent, when 80–90% of the bottom of the culture dish was covered with cells. The bone fragments were cut into 5–7 mm samples and were cultured in osteoblast medium containing Dulbecco’s Modified Eagles Medium (DMEM) (Gibco/Invitrogen, Carlsbad, CA, USA), Ham’s F-12 media containing 10% fetal bovine serum (FBS), and 0.1% penicillin/streptomycin (Thermo Fischer Scientific) [24].

### 2.5. Cultured Cell Treatment with Activated Serum

Upon reaching confluence, cultured cells were counted and replated at 20,000 cells/cm^2^ for twenty-four hours prior to treatment with AS. The control cells were also replated at the same density of 20,000 cells/cm^2^ with their appropriate culture medium for each cell line. Thus, tenocytes and bursal cells were plated in complete culture medium, chondrocytes were plated in chondrocyte medium, and osteoblasts were plated in bone medium. For cell treatment, five more 100 mL samples of whole blood were obtained (LAMPIRE Biological Laboratories Inc., Pipersville, PA, USA). As described above, AS was isolated from whole blood utilizing the Thrombinator. AS was diluted 1:1 in complete culture medium for the tenocytes and bursal cells, 1:1 in chondrogenic medium for chondrocytes, and 1:1 in bone medium for osteoblasts. The cells required complete culture medium in addition to AS in the experimental group because complete culture medium provides essential survival nutrients to the cultured cells. The cells receiving the AS were treated for one hour with 100 μL of AS. Based on a previous PRP study, this exposure time was chosen to mimic physiologic exposure that cells may experience with injection [25]. After one hour, AS was removed from the wells, and cells were washed three times with appropriate culture media. Then, 1 mL of appropriate media was added to each treated well and incubated at 37 °C. The control wells had 1 mL of appropriate culture media replaced.

#### Cellular Proliferation

Forty-eight hours after the addition of AS, the culture medium was removed from both experimental wells and control wells. A mixture of 600 μL of appropriate culture medium for each cell line and 300 μL of XTT-labeling mixture (sodium 3′-[1-(phenylaminocarbonyl)-3,4-tetrazolium]-bis (4-methoxy6-nitro) benzene sulfonic acid hydrate) were added to each well and allowed to incubate overnight at 37 °C. After incubating, the mixture was transferred to new wells. The absorbance was read on a BioTek^®^ (BioTek) plate reader at 450 nm with a reference wavelength of 650 nm. This assay forms a formazan dye through the cleavage of the tetrazolium salt, XTT, by active cells. As such, the absorbance detected by the plate reader functioned as a direct correlate to the number of actively dividing cells present in the culture.

### 2.6. Protein Expression

Enzyme-linked immunosorbent assays (ELISAs) were used to quantify the concentrations of established markers for inflammation released into the media surrounding the cells. This was performed to simulate the way in which AS alters the cellular response in musculoskeletal cell lines. Twenty-four hours following the treatment, the media were removed from the experimental and control wells. Samples were analyzed for growth factor expression, including platelet-derived growth factor (PDGF), vascular endothelial growth factor (VEGF), and insulin-like growth factor-1 (IGF-1), and for anti- and pro-inflammatory protein production, such as interleukin-1 beta (IL-1β) and interleukin-1 receptor antagonist (IL-1Ra).

### 2.7. Statistical Analysis

For statistical analysis, differences in cellular proliferation and protein expression were evaluated using a linear regression with a random intercept to account for repeated observation of the specimens across the testing conditions. The marginal means of the treated and control groups were compared to determine differences in proliferation and protein expression. A *p*-value of <0.05 was considered statistically significant. The analyses were performed using Stata statistical software (release 15 (2017); StataCorp, CollegeStation, TX, USA), except for the LC-MS analysis.

## 3. Results

### 3.1. Characterization of Activated Serum by LC-MS

In total, 357 peptides and 305 protein clusters were identified across the three samples of AS compared to whole blood (Supplemental LC-MS Data). The most abundant blood components that would crowd out the signal of the less abundant proteins of interest were depleted. The experiment utilized whole blood as a control to establish a baseline comparison of relative protein abundance in AS, expressed as a ratio (AS abundance: whole blood abundance). The 25 proteins with the largest ratio increase between AS and whole blood were identified and can be found in Table 1. These proteins were not identified in the whole blood sample, but were identified in the AS samples, making the change an infinite increase. Additionally, 14 of these 25 most common proteins were determined to have a statistically significant difference between their relative protein abundance (*p* < 0.05). Within the dataset, 54 of the proteins identified were found to have significant differences between the relative protein abundance when comparing the AS samples to the whole blood samples.

Additionally, based on the data in the Supplemental LC-MS Data, proteins of interest were identified across domains of growth, proliferation, and inflammation. Proteins of interest that were significant and detected within the growth and proliferation include, but were not limited to, insulin-like binding protein 3 (*p* < 0.05, 0.6-fold change) and protein S100-A6 (*p* < 0.05, infinite fold change). Proteins of interest within the domain of inflammation that were significant include, but were not limited to, catalase (*p* < 0.05, 11-fold change), superoxide dismutase (*p* < 0.05, 1.1E8 fold change), and neutrophil defensin-1 (*p* < 0.05, infinite fold change).

### 3.2. Cellular Proliferation

**Figure 1:**
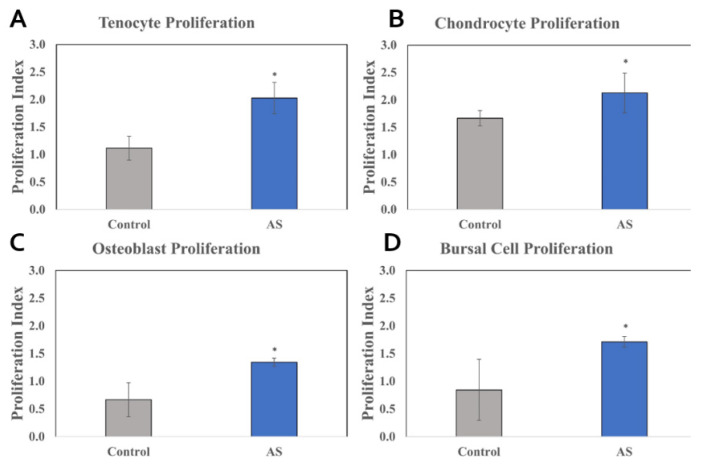
Cellular proliferation of cell lines treated with activated serum vs. control. XTT (sodium 3′-[1-(phenylaminocarbonyl)-3,4-tetrazolium]-bis(4-methoxy6-nitro) benzene sulfonic acid hydrate) assay analyzed the proliferation rate by measuring the absorbance of formazan salt at 450 nm. The absorbance value detected by the plate reader functioned as a direct correlate to the number of actively dividing cells present in the culture. The AS demonstrated higher rates of cellular proliferation compared to control in all cell lines. Tenocytes, chondrocytes, osteoblasts, and subacromial bursal cell lines can be observed above in (**A**–**D**), respectively. Statistical significance (*p* < 0.05) denoted by * (compared to control). AS, Activated Serum.

For all types of human cell lines tested (tenocytes, chondrocytes, subacromial bursal cells, and osteoblasts), cells treated with AS had significant increases in proliferation compared to control cells at 48 h. The proliferation of tenocytes exposed to the AS was increased compared to control (Δ = 0.92; *p* < 0.001; 95% CI: 0.71 to 1.14). For chondrocytes, the AS-exposed group was found to increase proliferation compared to control (Δ = 0.25; *p* = 0.044; 95% CI: 0.03 to 0.46). For the bursa cells, the AS-exposed group was found to increase proliferation compared to control (Δ = 0.81; *p* < 0.001; 95% CI: 0.60 to 1.03). For osteoblasts, the AS-exposed group was found to increase proliferation compared to control (Δ = 0.81; *p* < 0.001; 95% CI: 0.59 to 1.02). These results are shown in Figure 1.

### 3.3. Protein Expression

**Figure 2:**
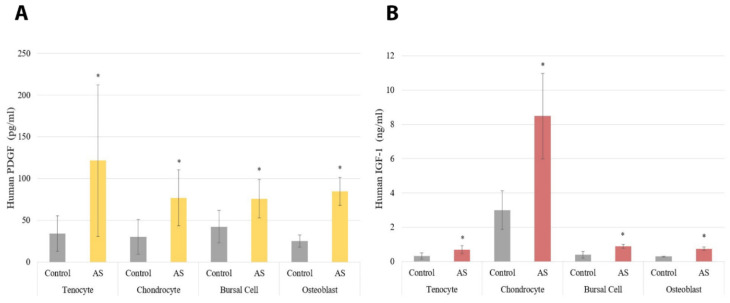
The protein expression of platelet-derived growth factor (PDGF) was significantly increased in all cells treated with activated serum compared to control, which can be observed in part (**A**). The protein expression of insulin-like growth factor 1 (IGF-1) was significantly increased in all cell lines treated with activated serum compared to control, which can be observed in part (**B**). Statistical significance (*p* < 0.001) denoted by *. AS, Activated Serum.

**Figure 3:**
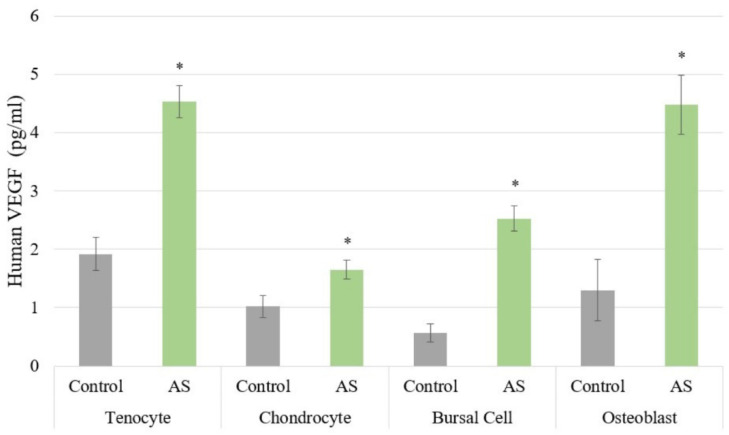
The protein expression of vascular endothelial growth factor (VEGF) was significantly increased in all cell lines treated with activated serum compared to control. Statistical significance (*p* < 0.001) denoted by *. AS, Activated Serum.

The results for the ELISAs are shown in Figure 2 and Figure 3. Cells treated with AS had increased PDGF protein expression compared to controls with a *p* < 0.001, regardless of cell type, as shown in Figure 2A. Similarly, all cells treated with AS had increased IGF-1 protein expression compared to controls with a *p* < 0.001 (Figure 2B). Moreover, VEGF protein expression was increased in all cells treated with AS compared to controls with a *p* < 0.001 (Figure 3). The pro-inflammatory cytokine IL-1β was not identified in any of the experimental samples or control groups. Lastly, IL-1Ra was detectable only in chondrocyte and bursa cells, with *p* = 0.708 and *p* = 0.441, respectively.

## 4. Discussion

The major finding of the study is that the addition of AS increases in vitro cellular proliferation and the release of growth factors that may play a role in tissue repair. The increased cellular proliferation of human tenocytes, chondrocytes, subacromial bursal cells, and osteoblasts was found to be statistically significant after treatment with AS compared to control after 48 h. Overall, these results exemplify that the AS promotes cellular proliferation in vitro, which may help play a functional role with tissue repair.

Another major finding in the study was that AS contains at least 357 peptides and 305 protein clusters that were identified across the three samples of AS compared to whole blood. Fourteen of the twenty-five most common proteins were determined to have a statistically significant difference between their relative protein abundance, where *p* < 0.05. This evidence supports that autologous thrombin systems may alter the composition of whole blood and may trigger the release of regenerative proteins to tissues.

Some of the proteins identified have been cited in previous literature to play a role in cellular proliferation. A few of the aforementioned proteins include protein S100-A6 and insulin-like growth factor binding protein 3. Together, these proteins have been implicated in the regulation of proliferation, apoptosis, and mitochondrial and cellular homeostasis [26,27]. Additionally, insulin-like binding protein 3 has also been shown to promote tissue regulation and wound healing in the cornea [28]. This evidence helps support that, given the difference in relative protein abundance among AS and whole blood samples, AS may provide an added benefit of containing a greater abundance of proteins that have been described to aid in cellular proliferation. Proteins of interest within the domain of inflammation include, but were not limited to, catalase, superoxide dismutase, and neutrophil defensin-1. These proteins are largely nonspecific inflammatory mediators that play a role in reducing oxidative stress, inflammation, and wound repair [29,30,31]. This evidence exemplifies that AS may provide an added benefit of wound repair through reducing oxidative stress and inflammatory mediators. However, future studies are required to complete a more in-depth analysis of AS and how proteins of interest may function to alter cellular proliferation and wound repair. A third major finding in this study was that the musculoskeletal cell lines also showed differing levels of growth factor production, as well as anti- and pro-inflammatory proteins, after being exposed to AS compared to control cells. The study found statistically significant increases in PDGF protein expression compared to controls in all cell lines. PDGF, whose activation stimulates cell growth and changes in cell shape and motility, has proven to be important during embryonic development and wound healing [32]. The function of PDGF and the finding of its statistically significant increase support that AS may help to promote wound repair. Additionally, VEGF protein expression was increased in all cells treated with AS compared to controls. VEGF induces endothelial cell proliferation, regulates angiogenesis, and promotes cell migration, which is also beneficial for healing tissue [33]. Cells treated with AS also had a statistically significant increase in IGF-1 protein expression compared to controls. IGF-1 has been shown to carry independent growth-stimulating effects that mediate linear growth and may optimize cartilage cell growth when combined with the synergistic action of growth hormone [34]. Lastly, IL-1β expression was not identified in any of the experimental samples or control groups. On the other hand, IL-1Ra was detectable only in chondrocytes and bursa cells, although there was no difference between the experimental and control groups. IL-1β is a proinflammatory cytokine that has been implicated in inflammation, pain, and autoimmune states, whereas IL1-Ra functions to inhibit its activity. With statistically significant increases in PDGF, VEGF, and IGF-1, these results help support that, when musculoskeletal cells are exposed to AS, there is an increase in the production of growth factors that have been shown to promote wound repair and tissue regeneration. Second, with no detection of IL-β, this indicates that the exposure to AS may not have induced inflammation in the cells. With only limited IL-Ra detection, there were no statistically significant differences between the treated cell lines and in the control. Thus, more research is necessary to analyze the reproducibility of this finding. In turn, these results may provide further evidence that AS may be an applicable option for biological augmentation of rotator cuff repairs, as it shows increased expression of pro-regenerative proteins in cell culture.

The emerging field of orthobiologics seeks to use substances, often naturally occurring in the patient’s body, to help injuries heal more quickly. A prothrombinase complex is generated by exposing whole blood to negatively charged glass beads and calcium chloride, activating thrombin to induce the platelet coagulation cascade [17]. The potential benefits of AAS are two-fold. First, the high concentration of thrombin within AAS allows for creating an autologous clot. Autologous clots can allow orthopedic surgeons to efficiently and locally deliver orthobiologics to a repair site that will not immediately be washed away by arthroscopic irrigation [35]. Secondly, as the present study demonstrates, AS contains many blood peptides beyond thrombin that may aid in tissue repair. The present study showed that in vitro exposure of cells to AS increased the proliferation and growth factor expression while not increasing deleterious cytokine expression. These results align with previous studies that suggest biological augmentation via cellular exposure to activated serum exposes tissues to a novel cytokine and signaling profile that may assist with healing [36].

Previous literature has described the results of exposing musculoskeletal tissue to growth factors. Leong et al. describes the necessary role that growth factors such as PDGF, VEGF, and IGF-1 have on tendon and ligament healing [37]. Muench, et al. found that, when IGF-1 is added to musculoskeletal cells, there is increased cellular proliferation over time [38]. Lastly, Liu et al. showed that there is a cellular proliferation benefit to growth factor treatments; however, there seems to be some variability in the efficacy of long-term treatments [39]. The current study has exemplified multiple areas of evidence supporting the current literature, with expanded evidence that the application of AS to musculoskeletal cells may be used to help promote musculoskeletal tissue repair, providing a promising future for biologic augmentation to musculoskeletal procedures.

This study is not without its limitations. First, this in vitro study may not replicate the in vivo interaction between the various cell types and AS. Therefore, more research is needed to evaluate the importance of AS for orthopedic biologic augmentation. Additionally, future studies are required for further investigation and analysis of the way in which AS alters the functional phenotypic outcome of the musculoskeletal cell lines. Second, the cell lines were obtained from different donors than the blood used to create AS; thus, it was not autologous as would be the case for intraoperative biologic augmentation. The use of autologous blood was not feasible for the present study, as multiple weeks were required to grow the cell lines for experimental purposes. Another potential limitation is that the study utilized an appropriate culture medium as a control and did not utilize whole blood as a baseline for how whole blood would affect cellular activity. Thus, the study may show a larger effect than is actually occurring due to the presence of baseline growth factors and anti- and pro-inflammatory proteins in whole blood as well. However, the goal of the study was to compare augmentation (AS treatment) to non-augmentation (control), which is why the culture medium was chosen as a control. Additionally, the samples that were run on the mass spectrophotometer were only run for an hour, so many less abundant proteins may have been below the detection limit and therefore not identified. Finally, the tissue obtained was discarded during orthopedic operations, and there was a lack of information on the demographics of the patients from which the samples were retrieved.

More research is necessary to help support the use of activated serum as a biological augmentation in orthopedic procedures. Future directions of this research can further investigate activated serum and compare autologous activated serum and their respective responses in order to assess whether there are differences in cellular proliferation and protein expression within native tissues. Another future study should investigate whether or not tissue response to biologic augmentation is influenced by demographic information such as age, sex, etc. This information was unavailable, and thus, the current study was unable to control for patient demographics. Lastly, future studies should investigate a similar research design with a larger sample size to help ensure the reproducibility of this study’s findings. Overall, this study provides promising evidence from cell culture data that can be used in future discoveries and investigations with regard to AS and its potential for biologic augmentation of rotator cuff repairs.

## 5. Conclusions

Serum activated through an autologous thrombin system may alter the relative protein abundance of whole blood. In human cell culture, AS increased the cellular proliferation and the release of human growth factors that may play a role in tissue repair compared to untreated control cells.

## Figures and Tables

**Table 1:** Summary table of 25 most relatively abundant proteins identified in activated serum compared to whole blood after depletion of major blood proteins, such as albumin, immunoglobulins (A, D, E, G, G-light chain, and M), alpha-1-acid glycoprotein, alpha-1-antitrypsin, alpha-2-macroglobulin, apolipoprotein A1, fibrinogen, haptoglobin, and transferrin. T-test compared the fold changes between the activated serum (AS) and whole blood samples. Infinite fold changes indicate that proteins in whole blood sample were below limit of detection.

Identified Proteins	Fold Change	*t*-Test (*p*-Value)
Spectrin alpha chain, erythrocytic 1	INF	<0.00010 *
Ankyrin-1	INF	0.00089 *
Purine nucleoside phosphorylase	INF	0.011 *
Methanethiol oxidase	INF	0.14
Band 4.1	INF	0.00016 *
Peroxiredoxin-6	INF	0.017 *
Phosphoglycerate kinase 1	INF	0.0014 *
Cluster of Nucleoside diphosphate kinase A	INF	0.00017 *
Transitional endoplasmic reticulum ATPase	INF	0.00016 *
Triosephosphate isomerase	INF	<0.00010 *
Parkinson disease protein 7	INF	0.00044 *
Protein 4.2	INF	<0.00010 *
Retinal dehydrogenase 1	INF	0.13
Adenylate kinase isoenzyme 1	INF	0.15
Rab GDP dissociation inhibitor beta	INF	0.12
Protein S100-A6	INF	0.004 *
Eukaryotic translation initiation factor 5A (Fragment)	INF	0.13
Transaldolase	INF	0.13
S-formylglutathione hydrolase	INF	0.12
Protein S100-A4	INF	0.068
GTP-binding nuclear protein Ran	INF	0.12
D-dopachrome decarboxylase	INF	0.13
Heat shock cognate 71 kDa protein	INF	0.12
Polyubiquitin-B	INF	0.0014 *
Myotrophin	INF	0.0056 *

* Statistically significant difference at alpha of 0.05.

## Data Availability

The data are not publicly available due to concerns over patient privacy. Data are available on request.

## Supplementary Materials

The following supporting information can be downloaded at: https://www.mdpi.com/article/10.3390/jcm11123442/s1, Supplemental Methods liquid chromatography-mass spectrophotometry (LC-MS) [21], Supplemental LC-MS Data.
