# Supplementary material for: Activated Serum Increases In Vitro Cellular Proliferation and Growth Factor Expression of Musculoskeletal Cells
Source: J Clin Med. 2022 Jun 15;11(12):3442. doi: 10.3390/jcm11123442 (PMC9225433; doi:10.3390/jcm11123442)
Supplement: Supplementary file 1 [file jcm-11-03442-s001.zip › Supplemental Methods liquid chromatography-mass spectrophotometry (LC-MS).pdf]

Soluble proteins isolated from whole blood or autologous proteins were subject to centrifugation for 15 minutes at 2200-2500rpm. Protein depletion using High-Select^{TM} Top 14 Abundant Protein Depletion Resin within mini-spin columns were then diluted with 0.1 M ammonium bicarbonate in water, pH 8. All Cys residues were subject to reduction and subsequent alkylation using 5 mM dithiothreitol in 0.1 M ammonium bicarbonate and 10 mM iodoacetamide in 0.1 M ammonium bicarbonate, respectively. Proteins were digested with Promega sequencing grade modified trypsin at a 1:20 enzyme:protein ratio for 16 hr at 37°C with constant shaking in a thermal mixer. The digestion was quenched by adding concentrated formic acid to yield pH 2.5. Proteolyzed peptides were desalted by Pierce C18 Peptide Desalting Spin Columns using manufacturer's instructions.

Desalted, tryptic peptides were subjected to analysis via ultra-high performance liquid chromatography coupled to tandem mass spectrometry (UPLC-MS/MS) using a Thermo Scientific Ultimate 3000 RSLCnano UPLC system coupled directly to a Thermo Scientific Q Exactive HF mass spectrometer. Peptides were initially loaded onto a 25 cm Waters BEH analytical column and gradient-eluted using a 60 min linear, reversed phase separation. Peptides were ionized directly into the Q Exactive HF using nanoelectrospray ionization and mass analyzed using a Top15 data-dependent acquisition method. Peptide and protein identification and quantification was performed using the MaxQuant software suite (v1.6.10.43)[1]and embedded Andromeda search engine by a search against the full Uniprot Homo sapiens reference proteome (UP000005640, accessed Nov 3, 2020, last updated Jun 29, 2020). Search parameters included a minimum of 5 amino acids/peptide, fixed carbamidomethyl Cys and the following variable modifications: oxidation of Met, protein N-terminal acetylation, peptide N-terminal Gln to pyro-Glu conversion, and deamidation of Asn and Gln . Remaining parameters were left at default values. All results were filtered to a 1% false discovery rate at the protein and peptide-spectrum-match levels using a decoy database search and consequently uploaded into Scaffold Q+S (v5, Proteome Software, Inc.) for data visualization and further analysis.

References

1. Cox, J.; Mann, M. MaxQuant Enables High Peptide Identification Rates, Individualized p.p.b.-Range Mass Accuracies and Proteome-Wide Protein Quantification. *Nat. Biotechnol.* **2008**, *26*, 1367–1372, DOI:10.1038/nbt.1511.
